# PARTIAL DISTAL DUODENECTOMY IN PATIENT WITH ADENOCARCINOMA

**DOI:** 10.1590/0102-672020240003e1796

**Published:** 2024-03-18

**Authors:** Héctor LOSADA, Norberto PORTILLO, Andrés TRONCOSO, Renato BECKER, Rocio VERA

**Affiliations:** 1 Hospital Hernán Henríquez Aravena, Surgery, Anesthesia and Traumatology Unit, Temuco, Araucanía, Chile;; 2 Universidad de la Frontera, Surgery, Anesthesia and Traumatology Unit, Temuco, Araucanía, Chile;; 3 Hospital Hernán Henríquez Aravena, Pathology Unit, Temuco, Araucanía, Chile.

**Keywords:** Adenocarcinoma, Duodenal Neoplasms, Surgical Oncology, Adenocarcinoma, Neoplasias Duodenais, Oncologia Cirúrgica

## Abstract

**BACKGROUND:**

Duodenal adenocarcinoma is a small percentage of gastrointestinal neoplasms, around 0.5%, and its treatment is based on resection of the tumor, classically by pancreaticoduodenectomy. In recent years, however, segmental resections of duodenal lesions, that do not involve the second portion or the periampullary region, have gained relevance with good surgical and oncological outcomes as well as the benefit of avoiding surgeries that can result in high morbidity and mortality.

**AIMS:**

To report a case of an elderly female patient with malignant neoplastic lesion in the third and fourth duodenal portion, non-obstructive, submitted to surgical treatment.

**METHODS:**

The technical option was the resection of the distal duodenum and proximal jejunum with preservation of the pancreas and reconstruction with side-to-side duodenojejunal anastomosis.

**RESULTS:**

The evolution was satisfactory and the surgical margins were free of neoplasia.

**CONCLUSIONS:**

Segmental resections of the duodenum are feasible and safe, offering the benefit of preventing complications of pancreaticoduodenectomies.

## RESUMO

**RACIONAL:**

O adenocarcinoma duodenal é uma pequena porcentagem das neoplasias gastrointestinais, em torno de 0,5%, e seu tratamento baseia-se na ressecção da massa tumoral, classicamente por pancreatoduodenectomia. Nos últimos anos, porém, as ressecções segmentares de lesões duodenais que não envolvem a segunda porção ou a região periampular têm ganhado relevância com bons resultados cirúrgicos e oncológicos e com o benefício de evitar uma cirurgia que pode apresentar alta morbimortalidade.

**OBJETIVOS:**

Reportar o caso de uma paciente feminina, idosa, com lesão neoplásica maligna na terceira e quarta porção duodenal, não obstrutiva, submetida a tratamento cirúrgico.

**MÉTODOS:**

A opção técnica foi a ressecção do duodeno distal e jejuno proximal com preservação do pâncreas e reconstrução com anastomose duodenojejunal látero-lateral.

**RESULTADOS:**

A evolução foi satisfatória e as margens cirúrgicas foram livres de neoplasia.

**CONCLUSÕES:**

As ressecções segmentares do duodeno são factíveis e seguras, com os benefícios de evitar as complicações das pancreatoduodenectomias.

## INTRODUCTION

Duodenal adenocarcinoma (DA) is a rare tumor that represents approximately 0.5% of gastrointestinal tumors, and more than 50% of adenocarcinomas of the small intestine^1^ ^,^ ^4^. Resection of the primary tumor is the only curative treatment for this disease, and the preference to perform a pancreatoduodenectomy (PDD) or segmental resection of the duodenum will depend on the location of the lesion and the proximal margin close to the duodenal papilla^3^. Although a lymph node resection in a PDD is described as a better solution, it implies major surgery with pancreaticobiliary anastomosis and the likelihood of a significant morbimortality. However, a duodenal segmental resection, with a proximal and distal negative margin for the neoplasm, will involve an enteral anastomosis without the bile duct or pancreatic resection, avoiding a more complex anastomosis^2^ ^,^ ^4^ ^,^ ^5^ ^,^ ^7^.

Concerning the clinical-epidemiological profile, DA appears more frequently in patients in the fifth and sixth decade of life, with no significant difference between the two genders. It is mainly located in the second duodenal portion, presenting as a periampullary tumor, with the first, third, or fourth portions being rare locations^1^ ^,^ ^2^ ^,^ ^4^ ^,^ ^5^.

The adjuvant therapy is currently under discussion, with preference for treatments used nowadays for colorectal cancer, to which DA is frequently compared^4^.

The objective of this study was to record a case of DA, located on images in the first duodenal portion, in which it was performed a segmental resection of the duodenum with oncological margins and the reconstruction of the intestinal transit with a duodenojejunal anastomosis.

### Clinical case

**Figure 1 -:**
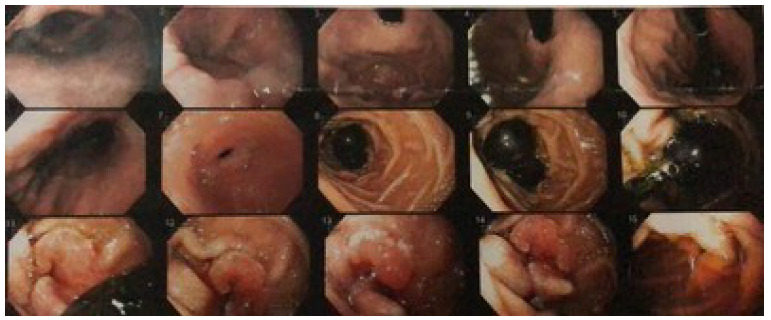
Upper endoscopy. Evidence of food remains extracted orally with Roth Net. In distal second duodenal portion, erythematous lesion with jagged edge is observed, that narrows the lumen.

**Figure 2 -:**
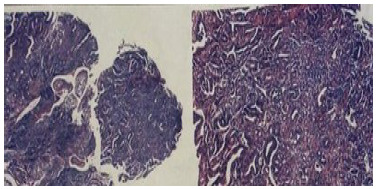
Biopsies from upper digestive endoscopy. Moderately differentiated papillary and tubular adenocarcinoma, infiltrating duodenal mucosa.

**Figure 3 -:**
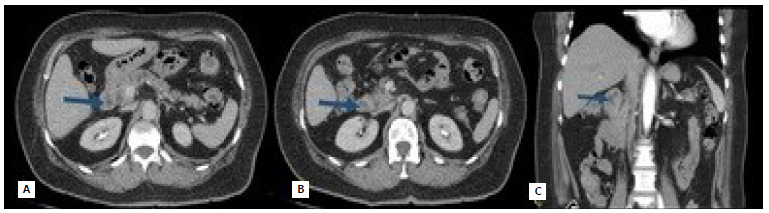
Computed tomography of abdomen and pelvis with contrast. A, B (axial section) and C (coronal section): concentric parietal thickening in the first portion of the duodenum with no signs of obstruction.

The study patient is a 70-year-old female with a history of arterial hypertension and dyslipidemia under good clinical control. Symptoms of abdominal pain began in the upper abdomen associated with anemia (hematocrit 29% and hemoglobin 9.6 g/dL). The renal, hepatic function tests, plasma electrolytes, proteinemia, and coagulation tests were within normal ranges, but presented hypercholesterolemia and hypertriglyceridemia. The upper digestive endoscopy (UDE) showed chronic atrophic gastritis with areas of metaplasia, and in the second duodenal portion, distal to the papilla, a stenosing erythematous lesion with jagged edges, accessible with difficulty (Figure 1). The biopsies revealed moderately differentiated papillary and tubular adenocarcinoma, infiltrating the duodenal mucosa (Figure 2). The colonoscopy indicated a sessile polyp in the descending colon and other small polyps in the sigmoid colon and rectum. All of them were resected, showing negative biopsies for neoplasm. Computed tomography (CT) evidenced a concentric parietal thickening of up to 11 mm in the first duodenal portion with an approximate extension of 6 cm displaying no signs of obstruction, as well as an increase in the density of the adjacent mesenteric tissue and some prominent regional lymph nodes of up to 12 x 10 mm in the hepatoduodenal ligament. In addition, the CT showed slight dilation of the bile duct up to 9 mm, and the common bile duct with progressive narrowing (Figures 3A, 3B, and 3C). With these findings, the patient was assessed by the hepatobiliary and pancreatic surgery team, which planned a PDD.

**Figure 4 -:**
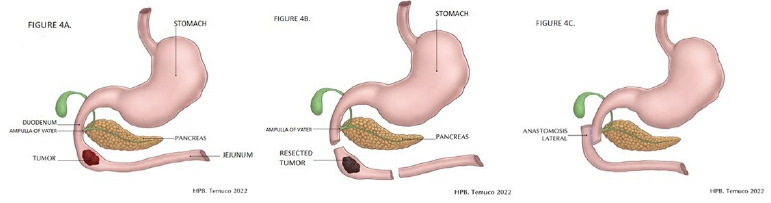
A. Duodenal tumor. Evaluation of ampulla of Vater involvement by intraoperative endoscopy; B. Duodenal tumor resection. Rapid biopsy showed negative margins; C. Latero-lateral duodenojejunal anastomosis.

An exploratory laparotomy was performed, and a Kocher maneuver was widened up to the intercavoaortic region, exhibiting the inferior vena cava and left renal vein. At this point, while the exploration was taking place, an indurated lesion was felt in the fourth duodenal portion, being marked with methylene blue using a fine needle. The decision was to conduct a simultaneous intraoperative UDE, which showed the neoplastic lesion in the fourth duodenal portion without involving the first, second, and third portions (Figure 4A).

**Figure 5 -:**
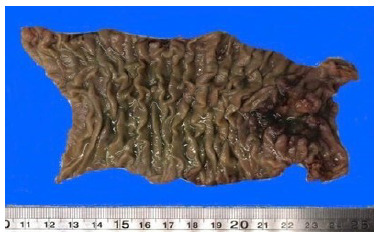
Macroscopy of surgical specimen (duodenum). Intestinal segment with ulcerated tumor lesion on the right end, with complete ring, and no involvement of margins (at 0.8 and 8.4 cm of edges).

The option was to perform a resection of the third and fourth duodenal portions with 10 cm of proximal jejunum (Figure 4B) and the reconstruction of the digestive tract with a latero-lateral duodenojejunal anastomosis (Figure 4C). The proximal margin sent for contemporary biopsy was reported as negative for neoplasm (Figure 5).

The postoperative period was uneventful and the evolution was favorable without complications. The drainage tube was removed on the fifth day and the patient was discharged on the seventh day with oral intake well tolerated.

**Figure 6 -:**
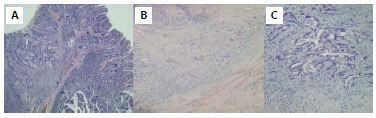
A. Histological section of tumor transition. On the right, mucosa of normal small intestine, in the middle, high grade dysplasia, and on the left, adenocarcinoma; B. Section of tumor area. Architecture distorted by proliferation of atypical gland-like elements, infiltrating the thickness of the intestinal wall; C. Tumor tissue with a tubular component, composed of poorly delineated structures, with the formation of sieves, made up of atypical cells with an increase in the nuclear-cytoplasmic ratio, anisokaryosis, hyperchromasia, and vesicular chromatin.

The histopathology revealed a moderately differentiated tubulopapillary adenocarcinoma, infiltration up to subserosal and adventitial fibroadipose tissue (Figures 6A, 6B, and 6C), proximal and distal edges without evidence of neoplasm, one lymph node resected without neoplasm, and a positive lymphovascular invasion.

The patient was referred to the Oncology unit and submitted to adjuvant treatment with capecitabine and oxaliplatin. She was in good condition, asymptomatic, and in well-tolerated chemotherapy at five months of follow-up.

The patient completed 16 months of follow-up, with UDE and CT presenting no evidence of neoplasm. She signed the informed consent form of the Institution for this publication.

### DISCUSSION

The standard surgery for duodenal neoplasm lesions is the PDD. However, it is possible to perform a segmental resection of a lesion when it is small and there is no involvement of either the ampulla of Vater or adjacent structures^1^ ^,^ ^2^ ^,^ ^5^. This method is preferred for distal lesions in the third and fourth portions.

The possible superiority of segmental resection over PDD in these cases has been suggested for several reasons, mainly due to the reduced complexity, that means performing a single enteral anastomosis compared to the three anastomoses (pancreatic, biliary, and gastrojejunal) of the classic Whipple^1^ ^,^ ^2^ ^,^ ^5^. In addition, it avoids greater potential morbidities (pancreatic fistula, biliary fistula, hemorrhage, or delay in gastric emptying).

Segmental resection of the lesion was performed despite planning a PDD due to the finding encountered intraoperatively: a lesion in the fourth duodenal portion with proximal safety margin suitable for this resection.

Some important technical aspects must be considered. The Kocher maneuver must be conducted until the entire duodenum is released and the fibers of the ligament of Treitz are released and dissected, employing intraoperative UDE in order to verify the relation with the major papilla, and ensuring oncological margins. It is also fundamental to identify the vascularization, so as not to compromise duodenal irrigation (superior mesenteric artery, inferior mesenteric vein, and gastroduodenal artery).

The reconstruction of the gastrointestinal tract after a segmental duodenal resection, employing the termino-terminal, latero-lateral, termino-lateral and latero-terminal duodenojejunal anastomoses has been described^2^ ^,^ ^5^ ^,^ ^6^. In the current case, we preferred the latero-lateral duodenojejunal anastomosis in a plane once in our team it is the standard technique applied in an enteral anastomosis. Besides, the anatomical presentation of the remnant duodenum facilitated the implementation of this technique.

Mitchell et al. described 83 duodenectomies with preservation of the pancreas, with results similar to those presented in the current case^5^. This surgery has likely increased given the oncological and surgical results described in the literature^1^ ^,^ ^2^ ^,^ ^5^. However, this technique must be proposed in cases where the surgical team has the skill to perform a conventional PDD, if required according to the intraoperative findings. In this case, the choice was for a segmental resection because the lesion had particular characteristics such as size, relation with the major papilla, and complete mobilization of the lesion.

### CONCLUSIONS

The segmental resection in duodenal lesions is feasible and a safe technique, and minimizes the risk of PDD-related morbidity.
